# APIP, an ERBB3-binding partner, stimulates erbB2-3 heterodimer formation to promote tumorigenesis

**DOI:** 10.18632/oncotarget.7802

**Published:** 2016-03-01

**Authors:** Se-Hoon Hong, Won Jae Lee, Young Doo Kim, Hyunjoo Kim, Young-Jun Jeon, Bitna Lim, Dong-Hyung Cho, Won Do Heo, Doo-Hyun Yang, Chan-Young Kim, Han-Kwang Yang, Jin Kuk Yang, Yong-Keun Jung

**Affiliations:** ^1^ School of Biological Science, Seoul National University, Gwanak-gu, Seoul 151-747, Korea; ^2^ Graduate School of East-West Medical Science, Kyung Hee University, Gyeoggi-Do 446-701, Korea; ^3^ Department of Biological Sciences, Korea Advanced Institute of Science and Technology, Daejeon 305-701, Korea; ^4^ Department of Surgery, Chonbuk National University Medical School, Jeonju 561-180, Korea; ^5^ Department of Surgery, Seoul National University College of Medicine, Seoul 110-744, Korea; ^6^ Department of Chemistry, College of Natural Sciences, Soongsil University, Seoul 156-743, Korea

**Keywords:** ERBB3, ERBB2, HRG-β1, gastric cancer, APIP

## Abstract

Despite the fact that the epidermal growth factor (EGF) family member ERBB3 (HER3) is deregulated in many cancers, the list of ERBB3-interacting partners remains limited. Here, we report that the Apaf-1-interacting protein (APIP) stimulates heregulin-β1 (HRG-β1)/ERBB3-driven cell proliferation and tumorigenesis. APIP levels are frequently increased in human gastric cancers and gastric cancer-derived cells. Cell proliferation and tumor formation are repressed by APIP downregulation and stimulated by its overexpression. APIP's role in the ERBB3 pathway is not associated with its functions within the methionine salvage pathway. In response to HRG-β1, APIP binds to the ERBB3 receptor, leading to an enhanced binding of ERBB3 and ERBB2 that results in sustained activations of ERK1/2 and AKT protein kinases. Furthermore, HRG-β1/ERBB3-dependent signaling is gained in APIP transgenic mouse embryonic fibroblasts (MEFs), but not lost in *Apip*^−/−^ MEFs. Our findings offer compelling evidence that APIP plays an essential role in ERBB3 signaling as a positive regulator for tumorigenesis, warranting future development of therapeutic strategies for ERBB3-driven gastric cancer.

## INTRODUCTION

Gastric cancer was once one of the most common cancer types and the second leading cause of cancer-related death in the world [1]. Whereas gastric cancer incidence has declined dramatically in Western countries, it still remains high in Eastern Asian countries [2]. Many genes have been analyzed in an attempt to understand gastric cancer pathogenesis and to improve clinical outcomes. Despite these efforts, only a few genetic changes, including the amplification and/or overexpression of MET and ERBB2 [3], as well as the mutations and downregulation of APC [4] and RUNX3 [5], have been associated with gastric cancer and the underlying molecular mechanisms remain largely undefined. Therefore, the identification of additional genes critical for the development and maintenance of gastric cancer is required.

The ERBB family plays an important role in normal embryonic development, angiogenesis [6], metastasis [7], cell proliferation [8] and apoptosis resistance [9]. Their aberrant signaling pathways are often found in human cancers [10, 11]. Among the ERBB family members ERBB2 receptor tyrosine kinase (RTK) is a pivotal driver of tumorigenesis [12, 13], and an important prognostic factor for patients with gastric cancer [14–16]. ERBB3 is unique as its intracellular tyrosine kinase domain is considered inactive or weak [17, 18], mainly gaining elevated activity through hetero-dimerization with other members of the ERBB family [17, 19]. Upon ligand-binding, these receptors homo-dimerize or hetero-dimerize, leading to their autophosphorylation and subsequent activation of the downstream phosphatidylinositol 3-kinase (PI3K). These mechanisms lead to mitogen-activated protein kinase (MAPK) signaling cascades [12]. The complex formed by orphan ERBB2 receptor and functionally weak ERBB3 has been recognized as being one of the most mitogenic signaling complexes in cancer development and maintenance [13, 20]. Therefore, better understanding the molecular mechanism underlying their activation through dimerization is key.

APIP has been associated with various types of cell death processes. The direct binding of APIP to APAF-1 highly inhibits mitochondria-mediated apoptosis [21]. During hypoxia, APIP reduces significantly hypoxic cell death by inducing the sustained activation of AKT and ERK1/2 in muscle cells [22]. It was recently shown that APIP inhibits caspase-1-mediated pyroptosis in response to *Salmonella* infection through its role within the methionine salvage pathway [23]. However, even though there are indications that the level of APIP is elevated in gastric tumor compared with normal tissues (www.proteinatlas.org) [24, 25], its role in tumorigesisis is unknown. APIP (located in chromosome region 11p13) amplification has also been observed in gastric cancer cell lines [26] and gastric cancers [27–29]. Finally, APIP is altered in non-small cell lung carcinoma (NSCLC) tumor [30]. In this study, we reveal a novel oncogenic function of APIP, which stimulates gastric cell proliferation and tumorigenesis through its interaction with ERBB3.

## RESULTS

### APIP is upregulated in human gastric cancers and cell lines

**Figure 1:**
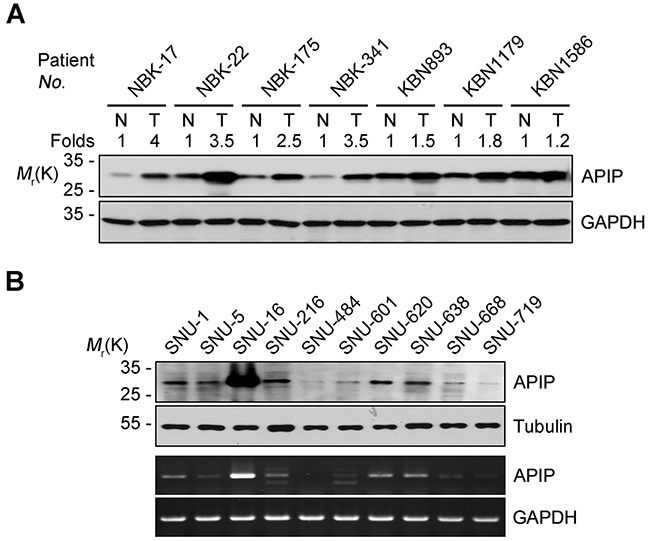
APIP is overexpressed in human gastric cancer cells and tissues **A.** Upregulation of APIP expression in human gastric cancer tissues. Cancer tissues and paired non-cancerous normal tissues of gastric cancer patients were analyzed by Western blotting. N, normal tissue; T, tumor tissue. **B.** Expression levels of APIP in human gastric cancer cell lines. APIP protein (upper) and mRNA (lower) levels were evaluated by Western blotting and RT-PCR analysis, respectively.

To characterize the role of APIP in gastric tumorigenesis, we examined APIP expression in human gastric cancer tissues. A total of 110 pairs of human gastric cancer tissues and adjacent gastric mucosa were examined in this study. Western blot analysis revealed an increased expression of APIP in 29 (26.4%) samples out of all gastric cancer tissues (Table 1). Among these 29 samples, the results from western blotting of 7 representative samples are shown in Figure 1A. Moderately and poorly differentiated tumors were associated with APIP expression (*P* = 0.039). However, there were no statistically significant correlations between APIP expression and histology, TNM stage or lymphatic invasion (Table 1). When we further assessed the clinicopathological and prognostic roles of APIP expression in human gastric tissues using immunohistochemistry (IHC), we observed a strong staining of APIP in gastric adenocarcinoma specimens, compared to normal samples (data not shown). We also evaluated APIP mRNA and protein levels in a panel of human gastric cancer cell lines (SNU-1, -5, -16, -216, -484, -601, -620, -638, -668 and -719) [31]. Most human gastric cancer cell lines expressed APIP but highly metastatic SNU-16 cells showed the highest expression of them all (Figure 1B).

**Table 1:** Correlation between APIP expression and clinicopathological characteristics of 110 gastric tumors cases

Tumor tissue parameters	n*	APIP expression		*P* value
		High (%)	Low (%)	
**All**	110	29 (26.4)	81 (73.6)	
**Gender**				ns
Male	79	20 (18.2)	59 (53.6)	
Female	31	9 (8.2)	22 (20)	
**Age (years)**				ns
Average, Range	63.4,32-85			
<55	25	6 (5.5)	19 (17.3)	
≥55	85	23 (20.9)	62 (56.3)	
**WHO classification**				0.039
W/D	13	3 (2.7)	10 (9.1)	
M/D	44	14 (12.8)	30 (27.3)	
P/D	45	11 (10)	34 (30.9)	
SRC	8	1 (0.9)	7 (6.3)	
**Lauren classification**				ns
Intestinal	102	26 (23.6)	76 (69.1)	
Diffuse	8	3 (2.8)	5 (4.5)	
**TNM stage**				ns
I	30	3 (2.7)	27 (24.5)	
II	37	9 (8.1)	28 (25.5)	
III	33	10 (9.1)	23 (20.9)	
IV	10	7 (6.5)	3 (2.7)	

Abbreviations: W/D, well differentiated tubular adenocarcinoma; M/D, moderately differentiated tubular adenocarcinoma; P/D, poorly differentiated tubular adenocarcinoma; SRC; signet ring cell carcinoma; ns, not significant.

* Numbers of cases in each group.

### APIP stimulates cell proliferation and gastric tumor growth

**Figure 2:**
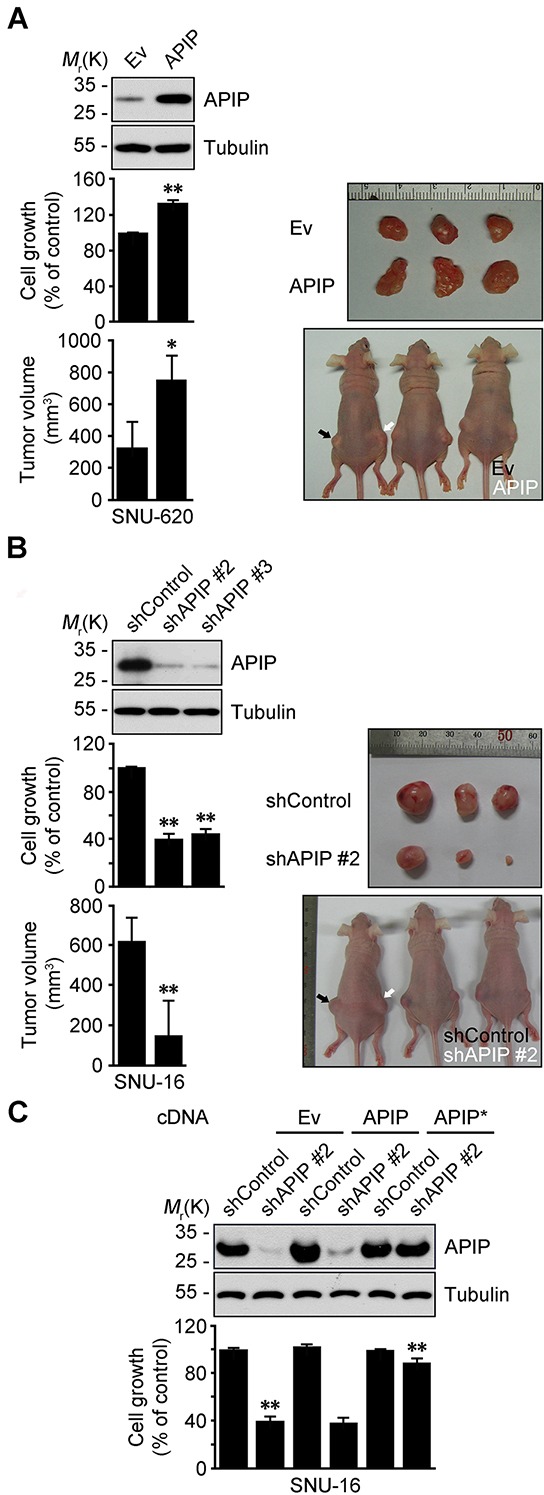
APIP positively regulates cell growth and tumorigenic potential in gastric cancer **A.** Overexpression of APIP in SNU-620 gastric cancer cells enhances cell proliferation (middle, *n* = 3) and promotes tumor growth (bottom, *n* = 5). Ev, pcDNA3 empty; APIP, pAPIP. Representative xenograft tumors of sacrificed mice (right). **B.** Downregulation of APIP expression in SNU-16 gastric cancer cells suppresses cell proliferation (middle, *n* = 3) and *in vivo* tumor growth (bottom, *n* = 5). shControl, pSUPER.neo; shAPIP #2 and #3, APIP shRNAs. **C.** Expression of shRNA-resistant APIP* rescues cell growth-inhibitory phenotype in SNU-16 APIP knockdown cells. SNU-16 control and APIP knockdown cells were transfected with pcDNA, pAPIP or pAPIP* (shRNA-resistant APIP) for 72 h. Cell growth rates (lower) and APIP protein levels (upper) were assessed. All data are represented as mean ± S.D. (*n* ≥ 3). Statistical significance is indicated as follows: *, *P* < 0.05; **, *P* < 0.01.

We then assessed whether APIP expression influences growth characteristics of gastric cancer cells. Cell proliferation was significantly enhanced by APIP overexpression in SNU-620 gastric cancer cells (SNU-620/APIP) (Figure 2A). Furthermore, xenograft assays in nude mice revealed a dramatic increase in tumor growth for APIP-overexpressing SNU-620 cells compared with control cells (Figure 2A). On the contrary, APIP knockdown suppressed the proliferation in SNU-16 gastric cancer cells (SNU-16/shAPIP #2 and #3) (Figure 2B; Supplementary Figure S1A to S1C). In agreement with cell line results, tumors derived from SNU-16 APIP knockdown cells grew substantially slower compared to those derived from SNU-16 control cells (Figure 2B). APIP knockdown itself did not trigger any cell death in SNU-16 cells (Supplementary Figure S1D). We confirmed that the observed shAPIP-mediated effect was specifically dependent on APIP knockdown by generating a shRNA-resistant APIP cDNA (APIP*); ectopic expression of APIP* cDNA in APIP knockdown cells restored the inhibitory effect of APIP shRNA on cell proliferation (Figure 2C). These results show that the regulation of APIP expression is crucial for cell proliferation and gastric tumor growth *in vivo*.

### APIP activates the AKT and ERK1/2 pathways for cell proliferation

**Figure 3:**
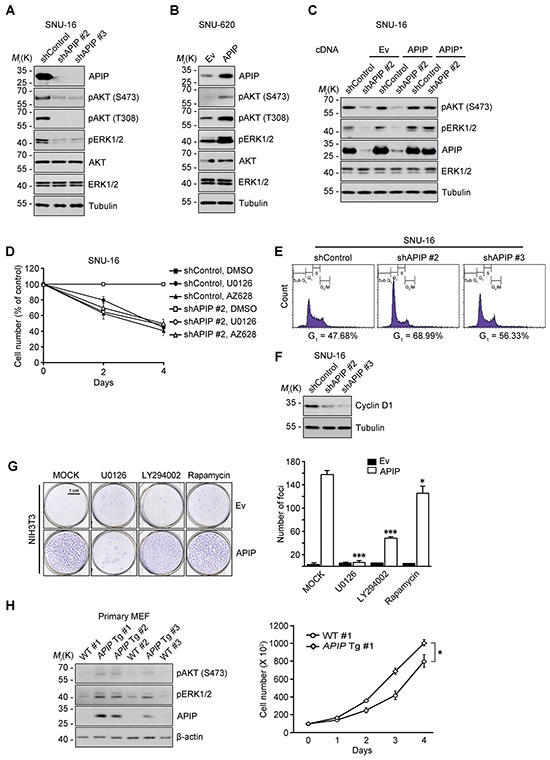
APIP affects both AKT and ERK1/2 pathways for cell proliferation **A.** and **B.** Knockdown or overexpression of APIP regulates AKT and ERK1/2 phosphorylation. Whole-cell extracts of SNU-16 control and APIP knockdown cells (A) or of SNU-620 control and APIP overexpression cells (B) were analyzed by western blotting. **C.** Reconstitution of APIP* rescues AKT and ERK1/2 phosphorylation in APIP knockdown SNU-16 cells. SNU-16 control and APIP knockdown cells were transfected with pcDNA, pAPIP or pAPIP* for 36 h. **D.** Effects of RAF1-MEK inhibition on cell proliferation in SNU-16 APIP knockdown cells. SNU-16 control and APIP knockdown cells were treated with 2 μM U0126 or 1 μM AZ628 for 4 days. **E.** Altered cell cycle distributions by APIP knockdown in asynchronous culture. SNU-16 control and APIP knockdown cells were stained with propidium iodide and analyzed by flow cytometry. Numbers indicates cell percentages at G_1_ phase. **F.** APIP knockdown decreases the expression level of cyclin D1. Asynchronous SNU-16 control and APIP knockdown cells were analyzed by western blotting. **G.** Overexpressed APIP exhibits focus-forming activity through MEK1/2 and PI3K. NIH3T3 control and APIP-overexpressing cells were assayed for focus formation with 2 μM U0126, 10μM LY294002 or 5 ng/ml rapamycin for 14 days, as visualized by crystal violet. Representative plates (left) and quantification of foci (right) are shown. Scale bar, 1 cm. **H.** Enhanced phosphorylation of AKT and ERK1/2 and cell growth in the primary *APIP* transgenic MEFs. Primary MEFs from wild-type (#1) and *APIP* transgenic (#1) were analyzed by western blotting (left) or maintained for 4 days to measure cell growth rate (right). All data are represented as mean ± S.D. (*n* = 3). Statistical significance is indicated as follows: **, *P* < 0.01. ***, *P* < 0.005.

We previously demonstrated that APIP sustains AKT and ERK1/2 activation under hypoxic condition in C2C12 mouse myoblast cells [21]. Therefore, we tested whether or not APIP stimulates cell proliferation via AKT and ERK1/2. In SNU-16 gastric cancer cells, APIP knockdown decreased the phosphorylation of AKT (Ser473 and Thr308) and ERK1/2 (Figure 3A). Inversely, APIP overexpression increased the activation of those pathways in SNU-620 cells (Figure 3B). As expected, overexpression of APIP* restored the activities of AKT and ERK1/2 in SNU-16 APIP knockdown cells (Figure 3C). In addition, APIP knockdown in SNU-16 cells reduced the reporter activity of c-Fos and Elk-1, downstream targets of ERK1/2 (Supplementary Figure S2A). These results indicate that APIP increases the activity of AKT and ERK1/2 in gastric cancer cells.

We then determined whether the AKT or ERK1/2 activation by APIP is responsible for the enhanced cell proliferation using kinase inhibitors. Treatment with PLX4032, a potent inhibitor of B-Raf^V600E^ in V600E-positive cells, or LY294002, a PI3K inhibitor, did not affect cell death or proliferation in SNU-16 cells. On the other hand, U0126, a MEK1/2 inhibitor, or AZ628, a RAF1 inhibitor, effectively suppressed cell proliferation without detectible induction of cell death (Figure 3D and Supplementary Figure S2B). This observation was similar to the suppression obtained through APIP knockdown in SNU-16 cells. There was no further additive effect of either U0126 or AZ628 on the inhibition of cell growth in APIP knockdown SNU-16 cells (Figure 3D), suggesting that APIP activates the RAF1 signaling pathway and ERK1/2 activation by APIP stimulates cell proliferation in SNU-16 cells. In addition, APIP knockdown increased the proportion of cells in the G_1_ phase by approximately 20% (Figure 3E) and resulted in the loss of cyclin D1 (Figure 3F), a key regulator involved in the G_0_/G_1_ phase transition. Furthermore, focus-formation assays revealed a dramatic increase in the number of foci in APIP-overexpressing cells (NIH3T3/APIP) compared with control cells (Figure 3G). However, the focus-forming activity of APIP was totally or partially disabled by U0126 and LY294002 respectively, while unaffected by rapamycin (Figure 3G).

To examine the potential role of APIP in cell proliferation *in vivo*, we generated APIP knockout mice and APIP transgenic mice which ubiquitously overexpress human APIP. An analysis of mouse embryonic fibroblasts (MEFs) showed a significant increase of cell proliferation rates as well as ERK1/2 and AKT activation in APIP transgenic MEFs compared with wild type MEFs (Figure 3H). On the other hand, APIP knockout had no obvious effect on cell proliferation in primary MEF cultures (Supplementary Figure S2C and S2D).

### Oncogenic function of APIP is independent of its enzymatic activity in methionine metabolism

**Figure 4:**
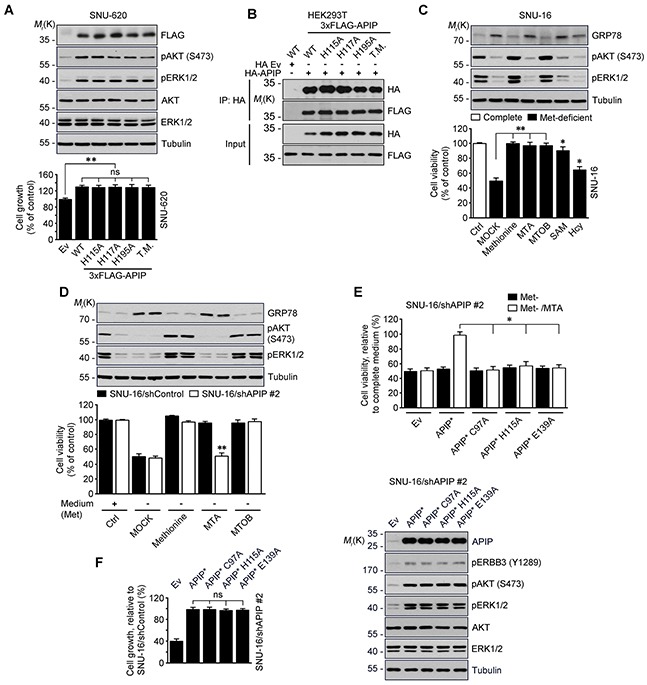
Evaluation of APIP/MtnB enzymatic activity in cell proliferation **A.** APIP/MtnB enzymatic activity is not associated with oncogenic function of APIP. SNU-620 cells expressing APIP (WT), single (H115A, H117A and H195A) or triple (T.M., H115/H117/H195A) mutants were measured for growth rates for 4 days. **B.** APIP mutants also form APIP/APIP multimers. HEK293T cells were co-transfected with 3xFLAG-tagged APIP (WT), single (H115A, H117A and H195A) or triple (T.M., H115/H117/H195A) mutant for 18 h. Whole-cell lysates were then subjected to immunoprecipitation (IP) assay. **C.** and **D.** Evaluation of APIP/MtnB enzymatic activity in SNU-16 cells. SNU-16 cells (C) or SNU-16 APIP knockdown cells (D) were maintained for 24 h in methionine-deficient media with the supplementations indicated. **E.** Reconstitution assay with APIP/MtnB mutants in methionine salvage pathway. SNU-16 APIP knockdown cells were transfected with the indicated APIP and incubated for 48 h in methionine-deficient media with 300 μM MTA. **F.** APIP/MtnB mutants display oncogenic activities in SNU-16 APIP knockdown cells. Cell growth rates (left) and APIP expression levels (right) were assessed. All data are represented as mean ± S.D. (*n* = 3). Statistical significance is indicated as follows: *, *P* < 0.05; **, *P* < 0.01; ns, not significant.

We examined whether APIP's methylthioribulose-1-phosphate dehydratase (MtnB) enzymatic activity is associated with its oncogenic function. As APIP is part of the zinc-dependent class II aldolase family and forms a tetrameric active enzyme in the protein crystal structure [32], we employed histidine mutants-H115A, H117A, H195A and their triple mutants with disabled enzymatic activity due to their incapacity to coordinates zinc ions. Like APIP, all of those APIP mutants stimulated cell growth with ERK1/2 and AKT activation (Figure 4A) and formed APIP/APIP multimers (Figure 4B).

Next, we checked the function of the methionine (Met) salvage pathway in SNU-16 cells. As previously reported for different cells [23, 33], deficiency of methionine in culture media induced endoplasmic reticulum (ER) stress and reduced cell viability in SNU-16 cells. However, when reintroducing precursors or intermediates of methionine salvage pathway, including 5′-methylthioadenosine (MTA), 4-Methylthio-2-oxobutanoic acid (MTOB), S-adenosyl methionine (SAM) and homocysteine (Hcy), cell viability was restored (Figure 4C). Unlike methionine and MTOB, MTA did not reduce the level of GRP78. Thus, this ER stress response was used as a control of methionine salvage pathway in the following step of the analysis. When we assessed the effects of methionine, MTA and MTOB on the survival of SNU-16 cells and SNU-16/APIP knockdown cells, we found that APIP deletion in SNU-16 cells impaired cell growth in the medium in which methionine was replaced by MTA (Figure 4D). Furthermore, reconstitution of SNU-16/APIP knockdown cells with APIP C97, H115 or E139 mutants, all of which are essential for MtnB activity in APIP, did not restore cell proliferation (Figure 4E). An assessment of the oncogenic activity of these mutants revealed that all of them stimulated cell proliferation as well as AKT and ERK1/2 activation, in a similar way with APIP in SNU-16/APIP* cells (Figure 4F). These findings suggest that the oncogenic function of APIP is dissociated from its enzymatic activity in the methionine salvage pathway.

### APIP enables HRG-β1-mediated ERBB3 activation

**Figure 5:**
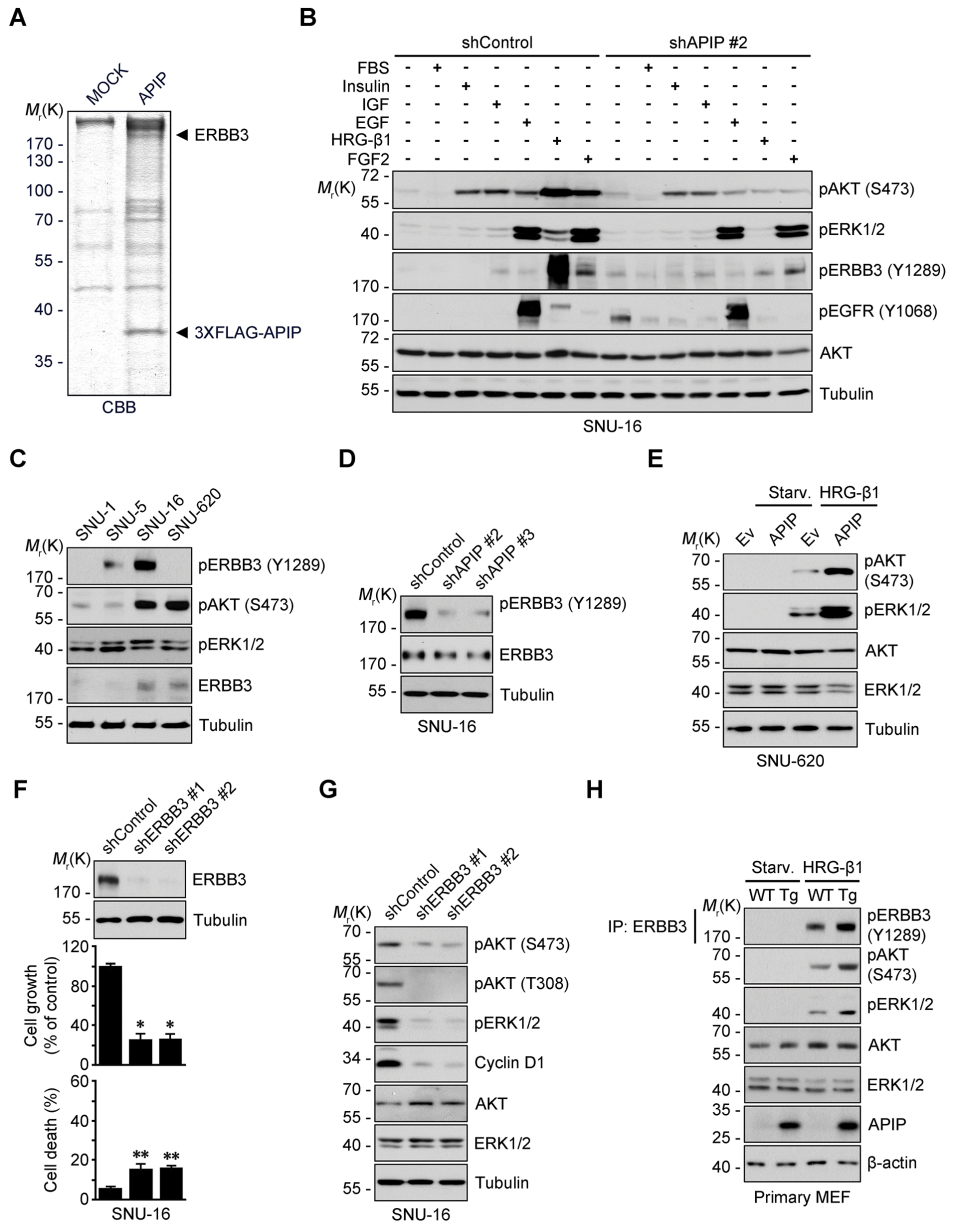
APIP is an essential activator of HRG-β1/ERBB3 in gastric cancer cells **A.** APIP-interacting proteins were purified from SNU-16 cells expressing 3xFLAG-tagged APIP by co-immunoprecipitation assay using FLAG M2 affinity gel. The bound proteins were resolved by SDS-PAGE and prepared for LC-MS/MS analysis. CBB, coomassie brilliant blue. **B.** Inhibition of HRG-β1-dependent ERBB3 phosphorylation and its downstream signals by APIP knockdown. Serum-starved SNU-16 control and APIP knockdown cells were treated with 10% FBS, 2 μM Insulin, 50 ng/ml IGF, 100 ng/ml EGF, 50 ng/ml HRG-β1 or 50 ng/ml FGF2 for 10 min and subjected to western blotting. **C.** Analysis of phospho-ERBB3 (Y1289) in a panel of gastric cancer cell lines by western blotting.**D.** Inhibition of ERBB3 activity by APIP knockdown in complete medium. Whole-cell extracts of SNU-16 control and APIP knockdown cells were analyzed with western blotting. **E.** APIP overexpression sensitizes SNU-620 cells to HRG-β1. SNU-620 control and APIP-overexpressing cells were stimulated with 2 ng/ml HRG-β1 and subjected to western blotting. Starv., serum starved. **F.** and **G.** ERBB3 knockdown inhibits cell growth and suppresses AKT and ERK1/2 activity. Cell growth (middle) and death rates (bottom) of SNU-16 control cells (shControl) or ERBB3 knockdown cells (shERBB3 #1 and #2) were assessed and analyzed by western blotting (top). The results represent mean ± S.D. (*n* = 3). (F). Whole cell lysates were examined by western blotting (G). **H.** Enhanced HRG-β1 signaling in *APIP* transgenic MEFs. WT and *APIP* transgenic MEFs were treated with 10 ng/ml HRG-β1 and harvested for immunoprecipitation (IP) assay. Statistical significance is indicated as follows: *, *P* < 0.05; **, *P* < 0.01.

To explore how APIP regulates AKT and ERK1/2 signaling in gastric cancer cells, we employed a pull-down assay using 3xFLAG-tagged APIP followed by a liquid chromatography-tandem mass spectrometry (LC-MS/MS) analysis. Interestingly, we found that ERBB3 is an APIP-binding partner (Figure 5A). We investigated the effects of several growth factors on the activation of RTKs and their downstream AKT and ERK1/2. Whereas insulin, IGF-1, EGF, HRG-β1 and FGF2 all activated ERK1/2 and/or AKT in SNU-16 cells, HRG-β1 was also a very potent stimulator of ERBB3 phosphorylation (Y1289), an activated form of ERBB3 [13] (Figure 5B). Assessment of ERBB3 expression in gastric cancer cell lines revealed high levels of phosphorylated ERBB3 (Y1289) in growing SNU-5 and SNU-16 cells but not in SNU-620 cells (Figure 5C). There was no difference in the levels of ERBB3 mRNA in these cells (Supplementary Figure S3A). Importantly, the activation of ERBB3, ERK1/2, and AKT by HRG-β1 was disabled by APIP knockdown in SNU-16 cells without affecting levels of ERBB3, EGFR or ERBB2 (Figure 5B and 5D; Supplementary Figure S3B). In addition, the HRG-1 β1-induced increase of pAKT, pERK and the reporter activity of c-Fos and Elk-1 were also impaired by APIP knockdown (Supplementary Figure S3C~SE). On the other hand, APIP overexpression enabled HRG-β1-induced activation of AKT and ERK1/2 in SNU-620 cells (Figure 5E and Supplementary Figure S3F). These results suggest that APIP is essential for the HRG-β1-induced activation of ERBB3 as well as for the downstream AKT and ERK1/2 pathways.

ERBB3 knockdown by shRNA inhibited cell growth while having a low incidence of cell death (Figure 5F), and generated a decrease in AKT and ERK1/2 activities (Figure 5G). Correspondingly, ERBB3 knockdown led to a significant decrease of cyclin D1 levels (Figure 5G). Overall, ERBB3 knockdown showed a very similar effect to APIP knockdown on both the delay in cell growth and inhibition of downstream signaling (Figure 5F and 5G). We further validated the potential role of APIP in the HRG-β1/ERBB3 pathway in APIP Tg and *Apip* knockout MEFs. In comparison with wild-type MEFs, APIP Tg MEFs showed an enhanced activation of ERBB3, AKT and ERK1/2 following HRG-β1 stimulation (Figure 5H). On the other hand, such differences in the activation of AKT and ERK1/2 between wild-type and *Apip* knockout MEFs in response to HRG-β1 could not be observed (Supplementary Figure S3G). These observations indicate that the gain-of-function of APIP in ERBB3 signaling is also present for the stimulation of cell proliferation in mice.

### APIP binds to the C-terminal tail of ERBB3

**Figure 6:**
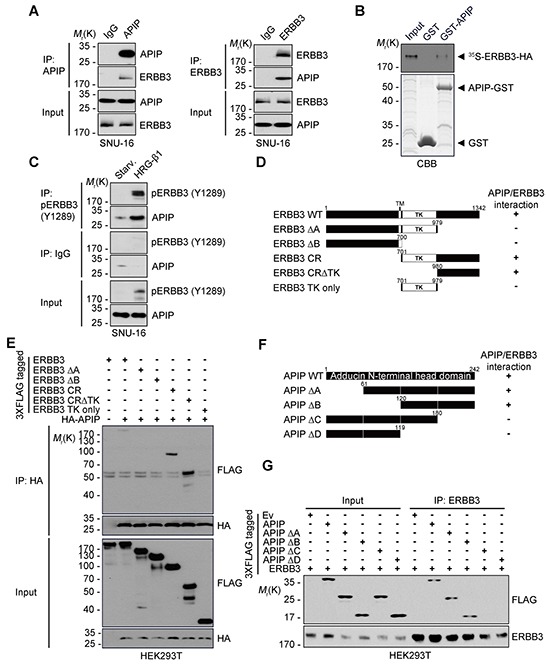
APIP interacts with ERBB3 via its C-terminus **A.** Co-immunoprecipitation of APIP and ERBB3 in SNU-16 cells. Whole cell extracts were subjected to immunoprecipitation (IP) assay with anti-APIP (left) or anti-ERBB3 (right) antibody. **B.** *In vitro* binding assay. *In vitro* translated and ^35^S-methionine-labeled ERBB3 protein was incubated with the purified GST or GST-APIP protein immobilized on glutathione-Sepharose 4B beads. The bound proteins were separated by SDS-PAGE and detected by autoradiography (upper) and CBB staining (lower). **C.** APIP binds to ERBB3 in a HRG-β1-dependent manner. SNU-16 cells were treated with 50 ng/ml HRG-β1 for 10 min and subjected to immunoprecipitation (IP) assay. **D.** Schematic representation of ERBB3 and its deletion mutants (Δ). The binding activities of ERBB3 WT and mutants to APIP are summarized based on the results in (E). **E.** Mapping of APIP-binding domain within ERBB3. HEK293T cells were co-transfected with pHA-APIP and either pERBB3-3xFLAG or deletion mutant for 24 h and whole-cell lysates were subjected to immunoprecipitation (IP) assay. **F.** Schematic representation of the conserved domains of APIP and its deletion mutants (Δ). The binding activities of APIP WT and mutants to ERBB3 are summarized based on the results in (G). **G.** The C-terminal region of APIP is essential for the binding to ERBB3. The immunoprecipitates were analyzed as in (E).

To gain insight into the molecular mechanism by which APIP regulates HRG-β1/ERBB3 signaling, we focused on the binding process between APIP and ERBB3. In proliferating SNU-16 cells, we detected ERBB3 in APIP protein complexes isolated by immunoprecipitation assay using an anti-APIP antibody and reciprocally (Figure 6A). We also confirmed the interaction between APIP and ERBB3 *in vitro* (Figure 6B). We evaluated the effect of HRG-β1 on this binding, showing that HRG-β1 treatment increased the binding of APIP to ERBB3 in SNU-16 cells, particularly to phosphorylated ERBB3 (Y1289) (Figure 6C and Supplementary Figure S4A). Alternatively, an investigation of the same binding process in a living cell using a split-Venus-based bimolecular fluorescence complementation (BiFC) assay enabled us to visualize the formation of ERBB3-VN/APIP-VC complexes in the plasma membrane and cytosol (Supplementary Figure S4B). The reverse combination of complexes (APIP-VN and ERBB3-VC) generated the same fluorescence pattern. Under this condition, co-expression of unlabeled full-length ERBB3 reduced the fluorescence in a dose-dependent manner (Supplementary Figure S4C), indicating that APIP specifically binds to ERBB3 in these cells.

To explore the binding regions in APIP and ERBB3 in greater detail, we generated serial deletion mutants of APIP and ERBB3 based on their secondary structures and reported domains (Figure 6D and 6F). The results of the immunoprecipitation assay revealed that APIP binds to the ERBB3-cytosolic region (CR), harboring a tyrosine kinase domain (TK, residues 701-979) and a C-terminal region (residues 980-1342), and the ERBB3-CRΔTK (residues 980-1342). However, APIP does not attach to the ERBB3-TK only or the ERBB3-ΔA lacking the C-terminal tail region (Figure 6D and 6E). These results suggest that APIP binds to the C-terminal tail region (residues 980-1342) of ERBB3. Similar assays revealed that APIP-ΔA and APIP-ΔB mutants lacking the N-terminal regions (residues 1-60 and 1-119, respectively) bind to ERBB3, whereas APIP-ΔC mutant lacking the C-terminal region (residues 181-242) did not interact with ERBB3 (Figure 6F and 6G), confirming the necessity of the C-terminal region of APIP for its binding to ERBB3.

### APIP enhances the binding of ERBB3 and ERBB2 in response to HRG-β1

**Figure 7:**
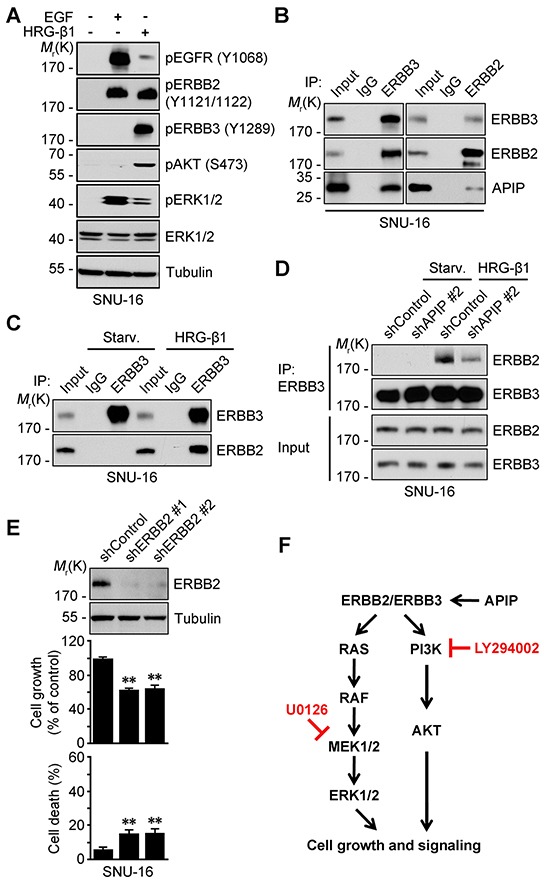
APIP stimulates the formation of ERBB3/ERBB2 heterodimer **A.** HRG-β1 induces the transactivation of ERBB2 in SNU-16 cells. SNU-16 cells were treated with 50 ng/ml HRG-β1 or 100 ng/ml EGF for 10 min and subjected to western blotting. **B.** APIP binds to ERBB3/ERBB2 complex in growing SNU-16 cells. SNU-16 cells were analyzed by immunoprecipitation (IP) assay with anti-ERBB3 (left) or anti-ERBB2 (right) antibody. **C.** HRG-β1 induces ERBB3/ERBB2 hetero-dimerization. SNU-16 cells were treated with 50 ng/ml HRG-β1 for 10 min and analyzed by immunoprecipitation (IP) assay. **D.** HRG-β1 induces ERBB3/ERBB2 interaction through APIP. SNU-16 control and APIP knockdown cells were treated with 50 ng/ml HRG-β1 for 10 min and analyzed as in (C). **E.** ERBB2 knockdown has a modest inhibitory effect on cell growth in SNU-16 cells. Cell growth (middle) and cell death (bottom) rates of SNU-16 control (shControl) and ERBB2 knockdown (shERBB2 #1 and #2) cells were assessed and whole-cell extracts were analyzed by western blotting (top). The results represent mean ± S.D. (*n* = 3). Statistical significance is indicated as follows: *, *P* < 0.05; **, *P* < 0.01. **F.** Proposed model of APIP/ERBB3/ERBB2 protein complex in cell growth signaling in gastric cancer cells.

ERBB3 requires hetero-dimerization with a suitable kinase-competent ERBB family member to activate HRG-β1/ERBB3 signaling. We first assessed the phosphorylation status of EGFR, ERBB2 and ERBB3 in serum-starved SNU-16 cells pretreated with EGF or HRG-β1 which differentially induces hetero-dimerization of ERBB family proteins [12, 34]. HRG-β1 mainly increased the phosphorylation of ERBB2 (Y1121/1122) and ERBB3 (Y1289) as well as AKT (S473) and ERK1/2 (Figure 7A), whereas EGF induced the phosphorylation of EGFR (Y1068), ERBB2 (Y1121/1122) and ERK1/2. We next examined whether APIP could stimulate the binding of ERBB3 with ERBB2, and thereby affect the activities of AKT and ERK1/2. A three-way immunoprecipitation assay showed that APIP formed a ternary protein complex with ERBB3 and ERBB2 (APIP/ERBB3/ERBB2) in growing SNU-16 cells (Figure 7B). On the other hand, there was no binding between ERBB3 and ERBB2 in serum-starved SNU-16 cells (Figure 7C). Furthermore, APIP knockdown in SNU-16 cells remarkably reduced the binding of ERBB3 with ERBB2, even in the presence of HRG-β1 (Figure 7D) or serum (Supplementary Figure S5). These results demonstrate that APIP increases the binding of ERBB3 to ERBB2 in response to HRG-β1. An evaluation of the contribution of ERBB2 to cell proliferation revealed that, as seen in ERBB3 knockdown, the loss of ERBB2 expression also suppressed cell proliferation in gastric cells (Figure 7E). However, this effect was lower than with ERBB3 knockdowns (Figure 5E). Overall, these results suggest that APIP stimulates cell proliferation via ERBB3, acting in conjunction with ERBB2, in gastric cancer cells (Figure 7F).

## DISCUSSION

In the present study, we report an important function of APIP in ERBB2/ERBB3 signaling in gastric cancer. Our findings provide APIP with an oncogenic function which had previously been suggested by reports showing increased expression or gene amplification of APIP in a variety of human cancers (early and advanced), including gastric cancer [27, 29]. The increased expression of APIP in gastric cancer cells enhances the HRG-β1/ERBB3-induced activation of AKT and ERK1/2 which stimulate cell proliferation and positively increase tumorigenesis by elevating ELK-1, C-FOS or cyclin D1 [35]. This signaling pathway is also conserved in other tumor cells, such as HeLa cervical carcinoma cells, as well as in MEFs. Therefore, the function of APIP in stimulating cell proliferation potentially operates in many cell types, including tumor cells and normal cells.

Then, how does APIP stimulate cell proliferation? It has been reported that ERBB3 alone is not sufficient to activate downstream signaling pathways because it is considered to be inactive and is classified as a pseudokinase [10]. It is thus conceivable to employ other ERBB receptors, such as ERBB2 or ERBB4, to activate ERBB3-mediated signaling pathway [13, 20]. ERBB4 was not detected in SNU-16 cells (Supplementary Figure S3H) and consequently not required for the amplification of ERBB3 signaling in SNU-16 cells. On the contrary, ERBB2 hetero-dimerized with ERBB3. As previously shown in EGFR [36], APIP binds to the cytoplasmic domain of ERBB3 to possibly stimulate dimer formation. Moreover, this formation of ERBB2/ERBB3 heterodimer was stimulated by APIP. As a model describing the role of APIP in stimulating the formation of this heterodimer, we suggest that APIP induces conformational change of ERBB3 after binding to it, resulting in an increase of the binding of APIP/ERBB3 to ERBB2 to stimulate heterodimer formation (Figure 7F). We also hypothesize that the binding of APIP and ERBB3 enhances the susceptibility of ERBB3 to HRG-β1 [37–39].

Another possible role of APIP in ERBB3 activation is receptor clustering, as shown in ligand-dependent receptor clustering [40]. A recent report showed that APIP exists as a tetrameric complex which is essential for the methionine salvage pathway [32]. In gastric cancer, APIP also forms a tetrameric complex and the inhibition of APIP/APIP interactions blocks its oncogenic activity. Thus, tetramer forms are important for the APIP oncogenic activity and might play a role in clustering its binding protein ERBB3. Because active forms of ERBB receptors are usually dimeric complexes and there is no direct interaction between ERBB2 and APIP, APIP can function as an enhancer of ERBB3 homo-dimerization or oligomerization [41]. In that sense, the presence of significant kinase activity of ERBB3 was recently emphasized. Although this activity of ERBB3 is weaker than the EGFR's, it is sufficient to phosphorylate its intracellular region and enable ERBB3-mediated*tran*s-autophosphorylation [18].

There are several known non-ERBB regulators of ERBB3, such as MET [42], BRK [43], EBP-1 [44] and CDK5 [45], in gastric cancer. All these non-ERBB regulators of ERBB3 directly phosphorylate ERBB3 [46] or recruit other protein(s) into the receptor complex [47]. APIP exhibits a similar activity, stimulating auto-phosphorylation of ERBB2 and ERBB3 as well as their activities. However, APIP is discriminated from other ERBB3-binding partners, such as c-Src [48, 49] and p85 subunit of PI3K [50] which also bind to the C-terminal regulatory (CR) domain of ERBB3, enabling its phosphorylation and activation in cancer cells. While these binding partners bind to ERBB3 through either their PTB or SH-2 domains [48, 49], there is no such domain in APIP. Indeed, APIP binds to ERBB3 through its uncharacterized domain. The possibility that APIP might form a protein complex with ERBB3 through binding to those ERBB3-binding partners or that it creates a signaling pathway along with non-ERBB regulators of ERBB3 cannot be excluded and needs to be further investigated.

In summary, this is the first report showing that APIP displays a pivotal oncogenic activity by binding to ERBB3 to stimulate the formation of ERBB3/ERBB2 heterodimer, leading to amplification of HRG-mediated signaling involved in cell proliferation and tumorigenesis in gastric cancer. Therefore, APIP can serve as a potential therapeutic target in gastric cancer, specifically targeting ERBB3 signaling in APIP-overexpressing cancers.

## MATERIALS AND METHODS

### Human gastric cancer tissues

Tissue samples from 110 patients with gastric cancer were studied after surgical resection at Chonbuk National University Hospital in Korea. The frozen fresh human tissue specimens and paraffin sections were supplied according to the regulation of the institutional review board (IRB). This study was approved by the IRB of Seoul National University (IRB No. 1009/001-002).

### FACS analysis

Cells were washed with ice-cold PBS and kept in 70% ethanol for one day for fixation. Fixed cells were washed with ice-cold PBS twice and incubated with propidium iodide (PI) (Sigma-Aldrich) solution (0.1% Nonidet P-40, 100 μg/ml RNase A and 200 μg/ml PI) for 10 min. Stained cells were analyzed by FACSCalibur™ flow cytometer (BD Biosciences), and the number of the cells in each stage was calculated with the ModFit LT™ cell cycle analysis program (Verity Software House) according to the manufacturer's instructions.

### Luciferase reporter assay

Cells cultured on 6-well plates were transfected with pFR-luciferase reporter plasmid and either pFA2-Elk-1-GAL4 or pFA2-C-Fos-GAL4 for 18 h. Whole-cell lysates were lysed in chemiluminescence lysis buffer and analyzed for luciferase activity with a luciferase assay kit (Promega) as recommended by the manufacturer. All luciferase activities were normalized by β-galactosidase activity.

### Bimolecular fluorescence complementation (BiFC) assay

Cells were transfected with VN or VC-fusion construct alone or in combination and incubated at 37°C for 18 h. After staining cell nuclei with Hoechst 33342, fluorophore formation in the living cells was imaged using a fluorescence microscope (Olympus, Hamburg, Germany) [51].

### Immunoprecipitation assay

For endogenous immunoprecipitation assay, 5 × 10^6^ SNU-16 cells were lysed in 600 μl CHAPS buffer containing 30 mM Tris-Cl pH 7.5, 150 mM NaCl, 1% CHAPS, 1 mM PMSF and 1 mg/ml each of aprotinin, leupeptin and pepstatin A. Whole-cell lysates were incubated for 12 h at 4°C with anti-APIP antibody or IgG after preclearance with 30 μl protein A/G–Bead (Santa Cruz Biotechnology). For co-immunoprecipitation assay, 1 × 10^7^ HEK293T cells were transfected with 3xFLAG-tagged APIP and HA-tagged APIP, and lysed in RIPA buffer (50 mM Tris-Cl pH 8.0, 15 mM NaCl, 1% Triton X-100, 0.5% sodium deoxycholate, 0.1% SDS, 1 mM PMSF and 1 mg/ml each of aprotinin, leupeptin and pepstatin A). Whole-cell lysates were incubated for 2 h at 4°C with monoclonal HA or FLAG antibody. After adding another 30 μl protein A/G–Bead, the mixtures were incubated for an additional 2 h at 4°C. The immunocomplexes were collected by centrifugation at 5,000 rpm for 3 min, washed 3 times with lysis buffer and detected by western blotting, as described above.

### Reverse transcription-PCR

Total RNA was purified using TRIZOL^®^ reagent (Invitrogen) according to the manufacturer's protocol. Reverse transcription was performed with a SuperScript II First-Strand Synthesis Kit (Invitrogen). PCR was performed using the following synthetic oligonucleotides sets: *GAPDH* (5′-GAA GGT GAA GGT CGG AGT CAA C-3′, 5′'-CCT GGA AGA TGG TGA TGG GAT T-3′); *APIP* (5′-CTG GGT CAC TGG GAC TGG AGG AGG-3′, 5′-GAA TAA AGA AAT GTA CTT CCG GAG GG-3′); *EGFR* (5′- ATG CGA CCC TCC GGG ACG GCC-3′, 5′-GAT AAG ACT GCT AAG GCA TAG G-3′; *ERBB2* (5′-CAC AGA CAC GTT TGA GTC CA-3′, 5′-AAA GCT CTC CGG CAG AAA TG-3′); *ERBB3* (5′-GGC AGC ACA CAG AGT TGC CC-3′, 5′-TCT CTG GGC ATT AGC CTT GGG G-3′); and *ERBB4* (5′-GGA AGA TAT GAT GGA TGC TGA GGA G-3′, 5′-TTT GGG TTT GTC TCG CAT AGG AG-3′).

### Plasmid construction

APIP cDNA was subcloned into the *Xho*1 site of pcDNA3-HA (pHA-APIP) or p3x-FLAG-CMV14 (3xFLAG-APIP). Empty vectors were used as control (Ev). The shRNAs were constructed using forward and reverse synthetic 19-nucleotides, which were synthesized, annealed, and sub-cloned into *Bgl*II and *Hin*dIII sites of pSUPER.neo. Sequences were as follows: shAPIP (#2, 5′-GGA CGG GAG TTT AAA ATT A-3′; #3, 5′-CCA TGT GTG AGT GTT ATG A-3′); shERBB2 (#1, 5′-TGG AAG AGA TCA CAG GTT A-3′; #2, 5′-GCC AGG TGG TGC AGG GAA A-3′); and shERBB3 (#1, 5′- GAG CGA CTA GAC ATC AAG C-3′;- AAG AGG ATG TCA ACG GTT A-3′).

### Complementation assay using shRNA-resistant APIP

APIP* that carries multiple silent mutations at the binding site of shAPIP #2 was constructed by site-directed mutagenesis using *pfu* DNA polymerase (Neurotics) and the oligonucleotide primer 5′-GGT CGC GAA TTC AAG ATA A-3′ (mutated residues are underlined).

### Cell culture

Human gastric cancer cell lines, SNU-1, -5, -16, -216, -484, -601, -620, -638, -668 and -719, were provided by Cancer Research Institute of Seoul National Hospital, Seoul National University [31]. Each cell line was maintained in a medium as follows: SNU cell lines in RPMI-1640 supplemented with 10% FBS (HyClone) and 2 mM glutamine; and MEFs, NIH3T3, HeLa and HEK293T cells in DMEM supplemented with 10% FBS. All cell lines were incubated in a humidified atmosphere of 5% CO_2_ at 37°C.

### Generation of *APIP* transgenic and *Apip* knockout mouse

A 729 bp fragment encoding human APIP cDNA (NM_015957.2) was subcloned into *Xho*I site of the mammalian expression vector pCAGGS [52]. The pCAGGS-APIP was digested with *Sal*I and *Hin*dIII to isolate the transgenic cassette consisting of CMV enhancer, chicken β-actin promoter, APIP cDNA and rabbit β-globin poly(A) sequence. The transgenic cassette was injected into mouse embryos with a C57BL/6 genetic background and four independent founders (F0) were identified by PCR analysis using synthetic oligonucleotides (forward, pCAGGS-for; reverse, APIP Tg-rev) (Macrogen, Seoul, Korea).

*Apip*/*Mmrp19* gene-trapped embryonic stem cells (XK742) were provided by BayGenomics of the International Gene Trap Consortium. Mice heterozygotes for *Apip* were generated by following the protocol provided by BayGenomics. Insertion of the gene-trap vector was confirmed by genomic PCR with a β-gal probe recommended by BayGenomics. The expression of APIP was examined using RT-PCR with synthetic oligonucleotides derived from *Apip* (*Apip*-5′ and *Apip*-3′) and β-gal. The absence of APIP expression in homozygous *Apip* KO (*Apip*^−/−^) mice was confirmed by Western analysis of the proteins extracted from the various tissues of *Apip* KO mice (4 weeks old). All animal experiments were performed with the approval of the Institutional Animal Care and use Committee of Seoul National University (IACUC No. SNU-120928-3). The animal treatments were carried out in accordance with the guidelines of the International Association for the Study of Pain (IASP). All mice were housed in an animal facility with a specific pathogen-free (SPF) barrier under a 12 h light/dark cycle.

### Primary culture of mouse embryonic fibroblasts

*APIP* Tg or *Apip* KO heterozygous mice were interbred to generate homozygous, heterozygous and wild-type embryos. Primary MEFs were derived from day 13.5 littermate embryo. The embryos were washed once with DMEM and 3 times with PBS. The tissue was then minced with a scalpel blade and digested with 0.01% Trypsin-EDTA (Invitrogen) for 30 min at 37°C. The tissue was shaken vigorously every 5 min during the incubation, and the mixture was then passed 3 times through an 18G needle to further dissociate any remaining clumps. Trypsin-EDTA was inactivated by addition of a 15-fold excess of DMEM containing 10% FBS. Cells from each embryo were put into 100 mm dish and cultured in DMEM with 10% FBS. Primary MEF from each embryo were plated separately and their genotypes were examined by Western blotting and genomic PCR. For standard culture condition, primary MEFs [passage (P) 2 or P3] were cultured in DMEM with 10% FBS.

### DNA transfection and reagents

Transfection was carried out using Polyfect^®^ reagent (Qiagen, Valencia, CA), Lipofectamine^®^ (Invitrogen) or PEI (Sigma-Aldrich) according to the manufacturers' instructions. Recombinant human HRG-β1 was purchased from R&D systems (Minneapolis, MN). EGF, IGF, FGF2 and insulin were obtained from Sigma-Aldrich.

### Generation of stable cell lines

SNU-620 and NIH3T3 cells were transfected with pcDNA3-HA or pHA-APIP for 24 h and then cultivated in selection medium containing 1 mg/ml G418 (Invitrogen) for 2 weeks. SNU-16 cells were transfected with pSUPER.neo (OligoEngine) or pAPIP shRNAs for 24 h and selected as above followed by western blot analysis.

### Cell death and cell proliferation assays

To determine cell death, cells were analyzed with trypan blue assay. To assess cell proliferation using DNA contents, 2.5 × 10^3^ cells were fluorometrically analyzed for their DNA contents with CyQUANT^®^ Cell Proliferation Assay kit (Molecular Probes) according to the manufacturer's instructions. Fluorescence was measured for excitation at 480 nm and emission detection at 520 nm on EnVision^®^ Multilabel Plate Reader (PerkinElmer, Wellesley, MA).

### Xenograft assay

Female athymic nude mice (4–5 weeks old) were obtained from the Central lab. Animal Inc. (Seoul, Korea) and injected subcutaneously with 2× 10^6^ cells in 0.1 ml of PBS containing 50 μl of Matrigel™ (BD Bioscience) into the flanks. Tumor volumes were measured weekly and calculated using the formula V=ab^2^/2 in millimeters, where ‘a’ is the length and ‘b’ is the width. Statistical analysis was performed by Student's *t*-test applied to the final time point.

### Focus-formation assay

Triplicate 6 well plates of confluent NIH3T3 control and APIP overexpression cells were left untreated or treated with a variety of inhibitors and cultured for additional 2 weeks. Media was replaced every 24 h thereafter until termination of the experiment. To inhibit focus formation, the inhibitors were added at every media change at the concentrations indicated. Cells were then washed with PBS, fixed with 10% formaldehyde and stained with 0.05% Crystal violet and the transformed foci were scored by manual counting.

### Western blotting and antibodies

Cells were lysed in a lysis buffer (50 mM Tris–Cl pH 7.4, 30 mM NaCl, 1% Triton X-100, 0.1% SDS, 1 mM EDTA, 1 mM PMSF, 1 mM Na_3_VO_4_, 1 mM NaF and 1 mg/ml each of aprotinin, leupeptin and pepstatin A) and sonicated briefly. The lysates were clarified by centrifugation, separated by SDS–PAGE and blotted onto nitrocellulose membrane (Millipore, Billerica, MA, USA). The membrane was blocked with 3% BSA in TBST (10 mM Tris-Cl pH 8.0, 150 mM NaCl, 0.5% Tween-20) and incubated overnight at 4°C with primary antibodies. The following monoclonal (m) and polyclonal (p) antibodies (Ab) were used: anti-APIP pAb (Imgenix); anti-PCNA mAb, anti-GAPDH mAb, anti-Tubulin mAb, anti-GFP pAb and Cyclin D1 mAb (Santa Cruz Biotechnology); anti-AKT pAb, anti-ERK1/2 pAb, anti-EGFR mAb, anti-ERBB2 mAb, anti-ERBB3 mAb, anti-AKT phospho (pS473) pAb, anti-AKT phospho (pT308) mAb, anti-ERK1/2 phospho (pT202 /pY204) mAb, anti-EGFR phospho (pY1068) mAb, anti-ERBB2 phospho (pY1221/pY1222) mAb and anti-ERBB3 phospho (pY1289) mAb (Cell signaling); and anti-FLAG mAb (Sigma-Aldrich).

### Statistical analysis

Data are expressed as mean ± S.D. Statistical comparisons between groups were performed using one-way analysis of variance (ANOVA) followed by Bonferroni's test or by the Student's *t*-test as appropriate. Probabilities of *P < 0.05, **P < 0.01 and ***P < 0.001 were considered statistically significant.

## SUPPLEMENTARY FIGURES


