# Supplementary material for: APIP, an ERBB3-binding partner, stimulates erbB2-3 heterodimer formation to promote tumorigenesis
Source: Oncotarget. 2016 Mar 1;7(16):21601–17. doi: 10.18632/oncotarget.7802 (PMC5008309; doi:10.18632/oncotarget.7802)
Supplement: Supplementary file 1 [file oncotarget-07-21601-s001.pdf]

SUPPLEMENTARY FIGURES

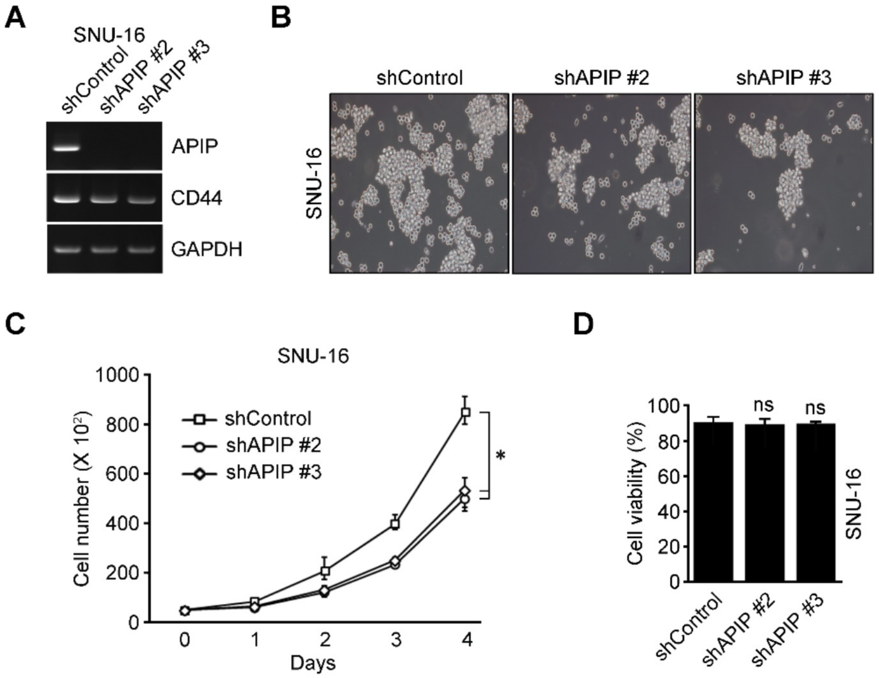

Supplementary Figure S1: APIP positively regulates cell growth and tumorigenic potential in gastric cancer. A. Determination of APIP mRNA levels in SNU-16 APIP knockdown cells. Total RNA samples of SNU-16 control and APIP knockdown cells were prepared and examined for APIP mRNA level by RT-PCR analysis using gene-specific synthetic primers. **B-D.** Downregulation of APIP in SNU-16 cells with shRNA suppresses cell growth. SNU-16 control and APIP knockdown cells were maintained for 4 days to measure the rates of cell growth (C) and cell death (D) and to take phase contrast micrographs at day 4 (B). The results represent mean ± S.D. (n = 3). Statistical significance is indicated as follows: *, $P < 0.05$.

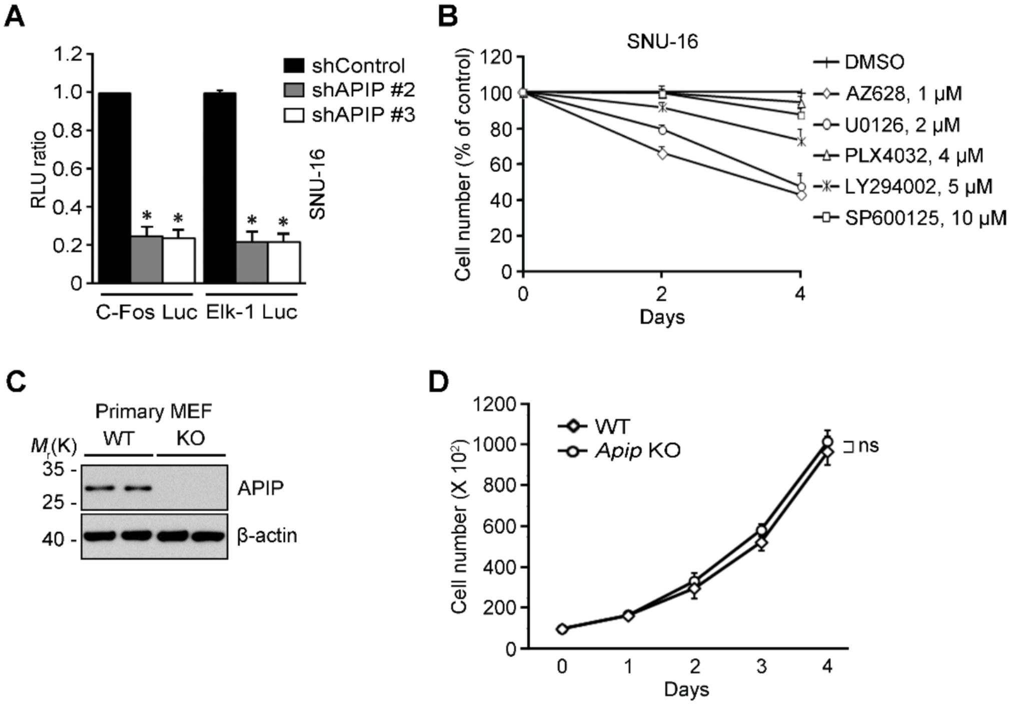

Supplementary Figure S2: APIP affects both AKT and ERK1/2 pathways for cell proliferation. A. APIP regulates Elk-1 and c-Fos activities in a reporter assay. **B.** Effect of RAF1-MEK inhibition on cell proliferation in SNU-16 cells. SNU-16 cells were left untreated or treated with the indicated concentrations of various inhibitors for 4 days. Cell growth rates were assessed with mean ± S.D. (n = 3). **C** and **D.** APIP depletion did not affect cell growth in primary MEF culture. Whole-cell extracts of MEFs were prepared and subjected to western blotting using anti-APIP antibody (C). Primary WT and APIP KO MEFs were maintained for 4 days to determine cell growth. Values represent mean ± S.D. (n = 3) (D). Statistical significance is indicated as follows: *, $P < 0.05$; ns, not significant.

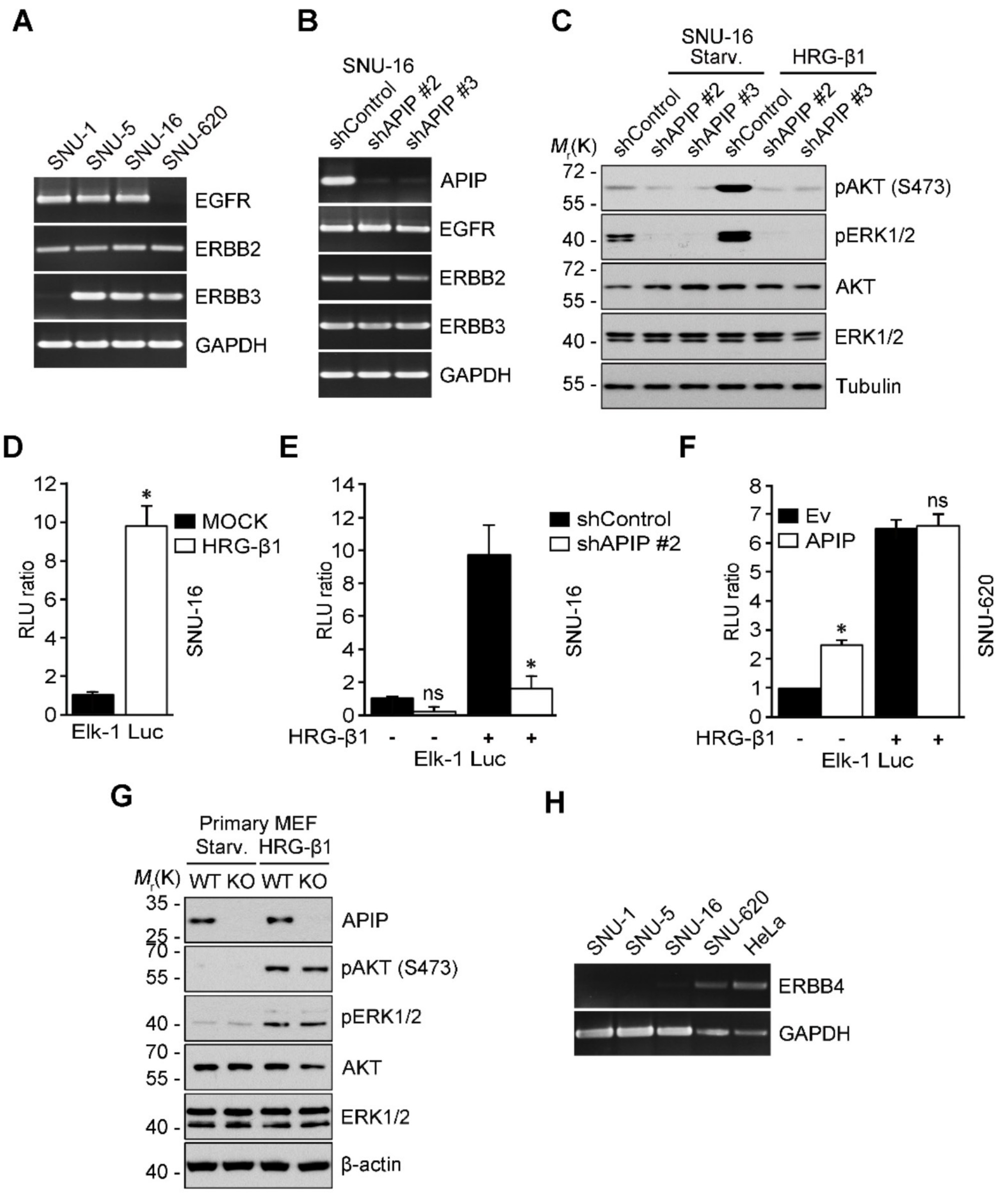

Supplementary Figure S3: APIP is an essential activator of HRG-β1/ERBB3 in gastric cancer cells. A. and **B.** Analysis of ERBB3, ERBB2 and EGFR mRNA levels in four gastric cancer cells (A) and SNU-16 APIP knockdown cells (B). Total RNA samples of gastric cancer cells were prepared and examined for the mRNA levels of EGFR, ERBB2 and ERBB3 by RT-PCR analysis. **C.** Stable knockdown of APIP expression by shRNA attenuates HRG-β1 signaling in SNU-16 cells. Serum-starved SNU-16 control and APIP knockdown cells were left untreated or treated with 50 ng/ml HRG-β1 for 10 min and harvested for western blotting. **D.** HRG-β1 positively regulates Elk-1 transactivation in SNU-16 cells. SNU-16 cells were co-transfected with a plasmid mixture containing of the Elk-1 luciferase-reporter system for 18h. Serum-starved cells were left untreated or treated with 50 ng/ml HRG-β1. After 1 h, the transfectants were lysed and assayed for luciferase activity. **E.** and **F.** APIP regulates HRG-β1-induced Elk-1 transactivation. SNU-16 control and APIP knockdown (E) or SNU-620 control and APIP overexpression (F) cells were transfected with the Elk-1 luciferase-reporter system for 18 h. Serum-starved cells were left untreated or treated with 50 ng/ml HRG-β1 for 1 h and luciferase activity was then monitored. Means of triplicate determinations ± S.D. are shown (n = 3). **G.** APIP depletion did not affect HRG-β1 signaling pathway in primary MEF culture. Serum-starved primary WT or *Apip* KO MEFs were left untreated or treated with 10 ng/ml HRG-β1 for 5 min and then harvested for western blotting. **H.** Analysis of ERBB4 mRNA levels in four gastric cancer cell lines. Total RNA samples of four gastric cancer cells (SNU-1, -5, -16 and -620) and cervical carcinoma cell (HeLa, a positive control) were prepared and examined for the mRNA level of ERBB4 by RT-PCR analysis. Statistical significance is indicated as follows: *, $P < 0.05$; ns, not significant.

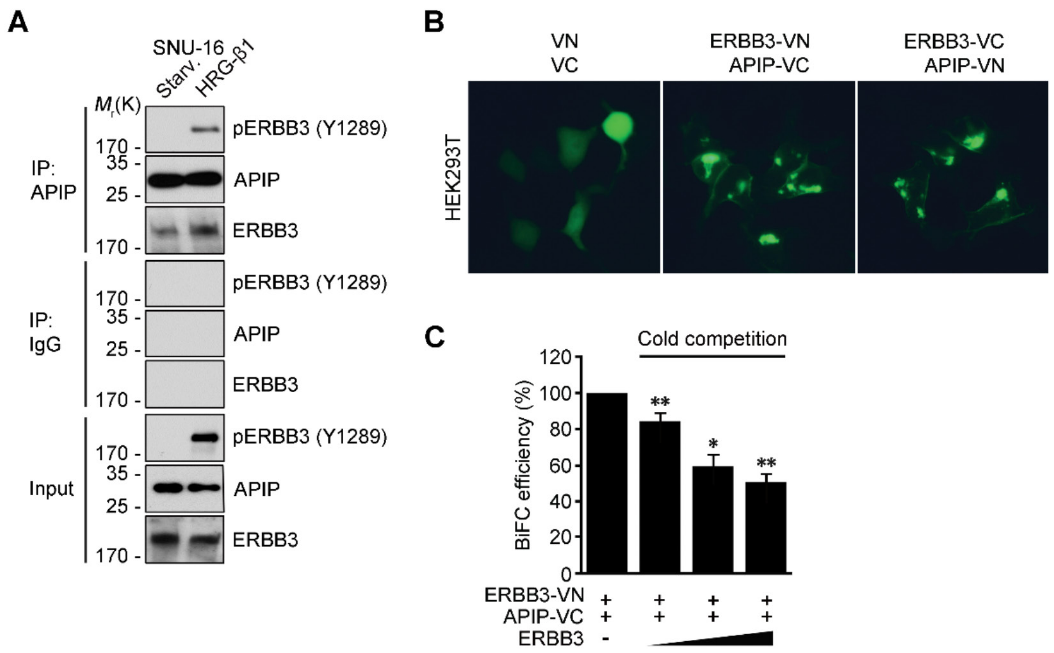

Supplementary Figure S4: APIP interacts with ERBB3 via its C-terminus. A. Protein-protein interaction between APIP and ERBB3. Serum-starved SNU-16 cells were left untreated or treated with 50 ng/ml HRG-β1 for 10 min and then subjected to immunoprecipitation (IP) assay. **B.** Visualization of the interaction of APIP with ERBB3 in living cells using BiFC analysis. HEK293T cells were co-transfected with equimolar amounts of pBiFC-VN and pBiFC-VC (a negative control), pERBB3-VN and pAPIP-VC or pAPIP-VN and pERBB3-VC for 12 h. **C.** Cold-ERBB3 decreases complementation fluorescence in a dose-dependent manner. HEK293T cells were co-transfected with pERBB3-VN, pAPIP-VC and increasing amounts of pERBB3 for 12 h. The complementation fluorescence was analysed under fluorescence microscope and quantified by counting number of fluorescence positive cells showing typical patterns. The results represent mean ± S.D. ($n = 3$). Statistical significance is indicated as follows: *, $P < 0.05$; **, $P < 0.01$.

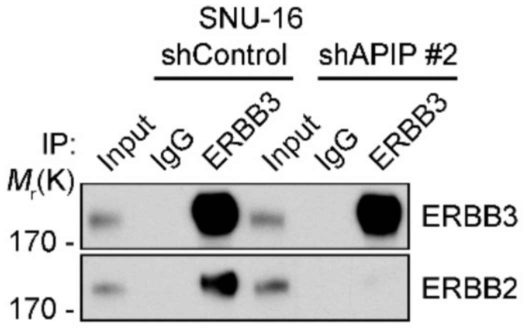

Supplementary Figure S5: APIP stabilizes the formation of ERBB3/ERBB2 heterodimer. Reduced association of ERBB3 with ERBB2 by APIP knockdown. SNU-16 control and APIP knockdown cells were subjected to immunoprecipitation (IP) assay with anti-ERBB3 antibody.
